# Unraveling the Mechanism of the 150-Fold Photocurrent Enhancement in Plasma-Treated 2D TMDs

**DOI:** 10.1021/acsami.2c06578

**Published:** 2022-07-18

**Authors:** Karolina Czerniak-Łosiewicz, Michał Świniarski, Arkadiusz P. Gertych, Małgorzata Giza, Zofia Maj, Maciej Rogala, Paweł J. Kowalczyk, Mariusz Zdrojek

**Affiliations:** † Faculty of Physics, Warsaw University of Technology, Koszykowa 75, 00-662 Warsaw, Poland; ‡ Faculty of Physics and Applied Informatics, University of Lodz, Pomorska 149/153, 90-236 Lodz, Poland

**Keywords:** two-dimensional materials, tungsten disulfide, transition metal dichalcogenides, plasma treatment, photocurrent enhancement, surface modification, optoelectronic memories

## Abstract

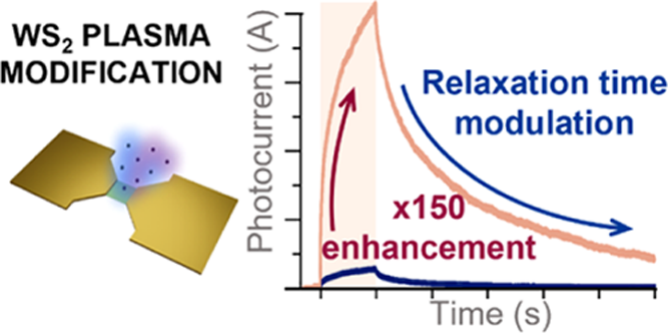

Two-dimensional (2D) transition metal dichalcogenides (TMDs) are increasingly investigated for applications such as optoelectronic memories, artificial neurons, sensors, and others that require storing photogenerated signals for an extended period. In this work, we report an environment- and gate voltage-dependent photocurrent modulation method of TMD monolayer-based devices (WS_2_ and MoS_2_). To achieve this, we introduce structural defects using mild argon–oxygen plasma treatment. The treatment leads to an extraordinary over 150-fold enhancement of the photocurrent in vacuum along with an increase in the relaxation time. A significant environmental and electrostatic dependence of the photocurrent signal is observed. We claim that the effect is a combined result of atomic vacancy introduction and oxide formation, strengthened by optimal wavelength choice for the modified surface. We believe that this work contributes to paving the way for tunable 2D TMD optoelectronic applications.

## Introduction

Transition metal dichalcogenide (TMD) monolayers have been extensively studied due to a direct band gap responsible for their favorable properties such as a high on/off ratio and efficient electron–hole generation.^1^ The optoelectronic properties of TMDs, especially molybdenum disulfide, are of particular interest because of the possibility of creating transparent and elastic photodetectors for wearable electronics.^2^ However, owing to the effects such as photogating and persistent photoconductivity, response times vary largely between reports, and often these materials show relatively slow response times.^3−5^ These properties could find use in some areas of optoelectronic applications, particularly those where the fast response is not vital.

In recent years, a new, interesting branch called neuromorphic engineering has gained a lot of attention, which aims to emulate the function of biological neurons to do computations.^6^ Neuromorphic sensors,^7^ optoelectronic synapses,^8^ and optoelectronic memories^7,9,10^ have been studied on 2D materials to perform computations such as image and pattern recognition, object detection, and storing sensitive information with light, which are all realized by maintaining an electrical signal for a specific time after illumination. In these applications, photogenerated carriers need to be trapped in the active material until they are processed as an electric signal for computational purposes. The mechanism of charge trapping after illumination is terminated, and maintaining the electrical signal is called persistent photoconductivity (PPC), seen previously in TMDs.^3^ However, different samples of TMDs show different times of maintaining PPC, from tens of seconds up to even ∼30 days.^11^

Several studies have shown TMDs undergoing plasma treatment to modify their properties. It has been reported that MoS_2_ photocurrent can be enhanced by oxygen plasma treatment, and the enhancement was a result of charge trapping at the heterojunction between molybdenum oxide (MoO*_3_*) and MoS_2_; however, it occurred only after a single plasma process, and the repetition of the treatment resulted in degradation of the photoresponse.^12^ WS_2_ samples were also previously subjected to plasma-induced defect formation. There have been reports of photoluminescence enhancement and patching up sulfur vacancies of WS_2_ by nitrogen plasma.^13,14^ The reduction of WS_2_ monolayer-based field-effect transistors’ (FETs) threshold voltage and mobility improvement were also seen after treatment with argon plasma due to the creation of sulfur vacancies in the WS_2_ layer and removing surface contaminants from the sample.^15^ Although some recent works on photocurrent in TMDs highlight the difference between measurements in different environments,^16,17^ none of the aforementioned plasma treatment experiments discussed the direct influence of plasma on photocurrent in relation to measurements performed in air and vacuum for WS_2_ and MoS_2_.

In the light of the aforementioned reports, we argue that the photocurrent enhancement of the TMD samples is a combined result of vacancy creation and oxide formation on the sample. In this work, we show on-chip tuning of the photocurrent response of WS_2_ to significantly enhance the electrical signal while elongating the duration of the PPC under UV illumination. The tuning effect is obtained by mild plasma exposure, which forms a few kinds of structures upon the sample: sulfur vacancies, nonstoichiometric transition metal oxide (TMO), and stoichiometric TMO. We show that the significant effect of photocurrent enhancement (over 150 times) stems from the optimized plasma process and the well-matched wavelength of the light used in our experiment that fits in the band-gap region of both TMDs and TMO. We also show that both oxide formation and the created vacancies in the sample increase the charge trapping mechanism.

Contrary to previous work on plasma-enhanced photocurrent generation in TMDs,^12^ we show the impact of the environment on the enhancing effect by employing photocurrent measurements in vacuum and air. Moreover, no photocurrent enhancement has ever been shown for WS_2_ monolayers under plasma treatment. No such high photocurrent increase has ever been shown for the plasma-treated monolayers of TMDs as well.

The plasma treatment and electrical measurements were also conducted for the MoS_2_ sample, and the results are consistent with WS_2_ measurements, suggesting the versatility of the modification method. Our work contributes to learning the tuning effect for creating optoelectronic devices that can be tailored to the desired properties, enabling multitudes of future applications, especially as optoelectronic memories or artificial optical synapses.

## Results and Discussion

**Figure 1:**
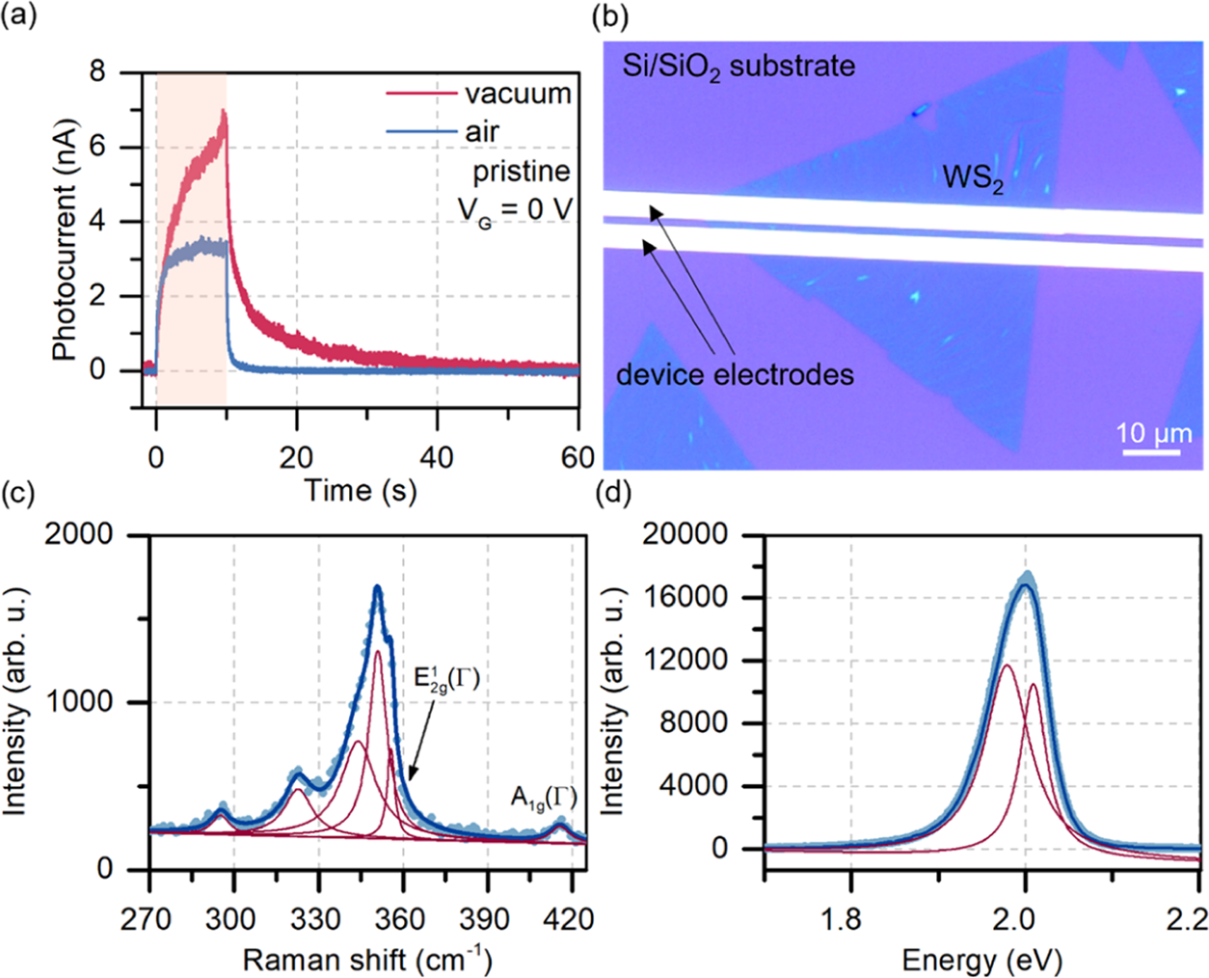
Initial characterization of the as-prepared WS_2_ sample. (a) Time-resolved photocurrent signal of the pristine WS_2_ sample in vacuum and air. The highlighted area corresponds to the time the device was illuminated. (b) Optical image showing the measured device. The channel was 2 μm long. (c, d) Raman spectrum (c) and photoluminescence spectrum (d) of the WS_2_ untreated sample were measured with a 532 nm laser. The spectra show the fitted Lorentzian functions for the specific peaks (red curves).

Pristine monolayer WS_2_ devices were first measured electrically in vacuum and air. The time-resolved photocurrent on devices based on WS_2_ in both environments was of the same order of magnitude, as shown in Figure 1. The rise and decay of the photocurrent followed the typical behavior of such devices—slower responses were observed in vacuum due to the lack of environmental adsorbates, which assist in relaxation.^18^ Raman and photoluminescence spectroscopy results and the optical image of the device are shown in Figure 1. The Raman spectra show typical Raman peaks of WS_2_ in resonance for a 532 nm laser. The observed feature is a combination of six peaks, including A_1g_ at ∼417 cm^–1^, E_^1^2g_ at 356 cm^–1^, and the most intense 2LA at 350 cm^–1^. Photoluminescence shows a highly intense peak typical for the monolayer material, fitted with two Lorentzian curves following the literature reports, showing the occurrence of neutral and negative excitons.^19,20^

**Figure 2:**
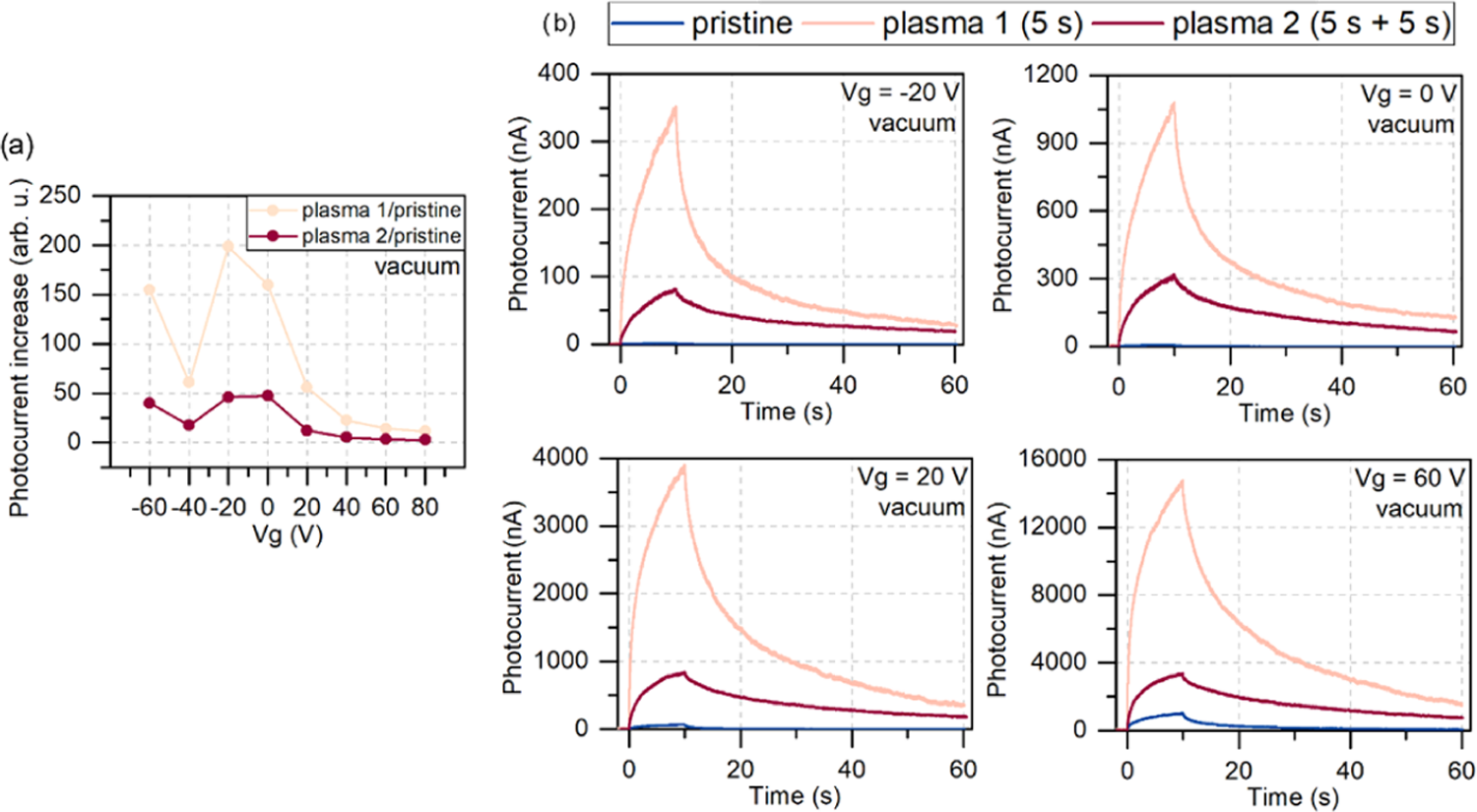
Comparison of the photocurrent measured in vacuum for treated and untreated samples. (a) Photocurrent increase calculated as a maximum photocurrent obtained after the same amount of time for each gate voltage and normalized by the maximum photocurrent of the pristine sample on WS_2_. The results show that the devices treated with a single plasma process respond stronger to illumination, and their response is the highest for low gate voltages. (b) The photocurrent signal of the samples before (pristine—blue lines) and after plasma treatment (first plasma—beige lines, second plasma—red lines) measured in vacuum. The graphs show the gate voltage dependence of the photocurrent for −20, 0, 20, and 60 V. Large enhancement of the photocurrent was observed after the first plasma treatment. The second plasma treatment also increased the signal compared to the pristine sample, but the effect was not as pronounced as for the first treatment. Although the point at −40 V results from a random unexpected event, the trend is visible.

Next, the samples underwent a plasma process (the details are in the Methods section). Two plasma processes were done in total on the samples. The first treatment was for 5 s (called plasma 1 in further text), and then the sample was measured in vacuum and air. Next, it was subjected to another 5 s of plasma treatment (plasma 2 in further text) and measured. Figure 2 shows the photocurrent measured on the WS_2_ sample before and after the first (5 s) and second (5 s + 5 s) plasma processes in vacuum under different gate biases. The applied gate voltages ranged from −60 to 80 V with a step of 20 V for the time-resolved photocurrent measurement.

We see that the plasma process significantly changed the photoresponse of the devices. After the first plasma treatment, we obtained over 150 times enhancement of the photocurrent value compared to the pristine sample of WS_2_ at zero gate bias (from 6.9 nA to 1.1 μA). It is a record value of photocurrent enhancement by plasma treatment in TMD monolayers. In vacuum, the dominating photocurrent signal comes after the first plasma process for both positive and negative gate bias. The second treatment decreased the signal to 50-fold enhancement compared to the pristine sample. We calculated the responsivity with the formula , where *I*_photo_ is the measured photocurrent and *P*_opt_ is the optical power of light. The values at zero gate bias for the samples pristine, after plasma 1, and after plasma 2 were 0.05, 166, and 6.5 mA/W, respectively.

**Figure 3:**
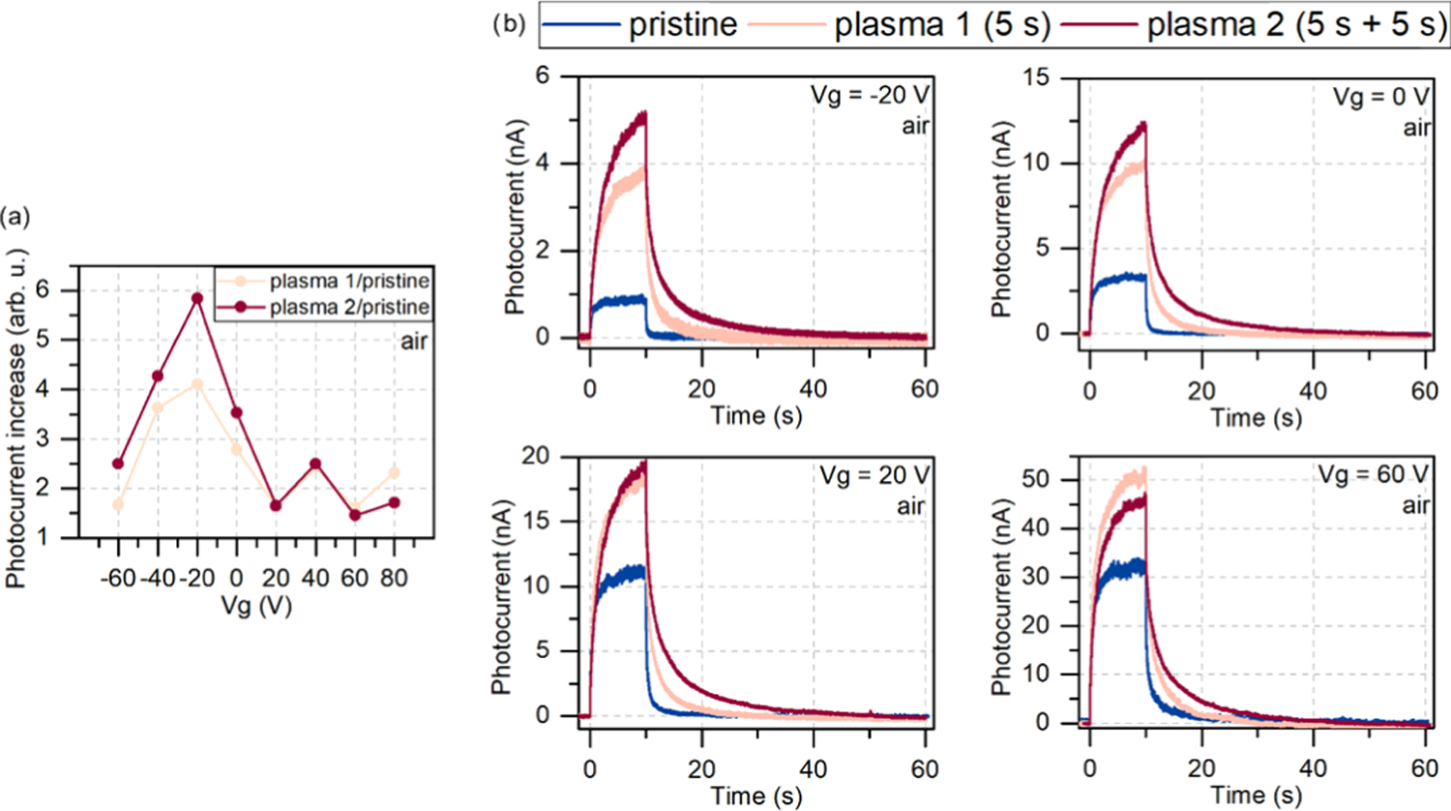
Comparison of the photocurrent measured in air for treated and untreated samples. (a) Photocurrent increase calculated as a maximum photocurrent obtained after the same amount of time for each gate voltage and normalized by the maximum photocurrent of the pristine sample in air. (b) The photocurrent signal of the samples before (pristine—blue lines) and after plasma treatment (first plasma—beige lines, second plasma—red lines) measured in air. The graphs show the gate voltage dependence of the photocurrent for −20, 0, 20, and 60 V. The photocurrent signal shows a stronger dependence on the applied voltage in air, where the dominating signal changes between the second and first plasma treatments. The highest photocurrent enhancement was observed for low gate voltages after two plasma processes. Still, with a high gate voltage applied, the first plasma treatment yields the best results in increasing the signal (here at 60 V).

The exact measurements were repeated for the sample in air and are shown in Figure 3. In the air, we also see the photocurrent enhancement; however, the signal strength behaves quite differently than that in vacuum.

Surprisingly, the photocurrent enhancement in air is much less impressive (below 3 and 3.5 times at zero gate bias for the first and second treatment, respectively), but it shows a gate voltage dependence. For negative and relatively low gate voltages (up to 20 V), the second plasma treatment resulted in a dominating photocurrent signal. This changes at a gate bias of 40 V when both signals are almost equal, and for higher gate voltages, the dominating one is the signal after the first plasma treatment. To the best of our knowledge, such intriguing dependence of photocurrent enhancement on the environment and gate voltage has never been explored. Similar behavior of the MoS_2_ samples is shown in Figures S1 and S2. A complete comparison between photocurrents measured for WS_2_ at all applied gate voltages is shown in Figure S3. The responsivity values calculated at zero gate voltage for the samples pristine, after plasma 1, and after plasma 2 were 25, 77, and 92 μA/W, respectively.

**Figure 4:**
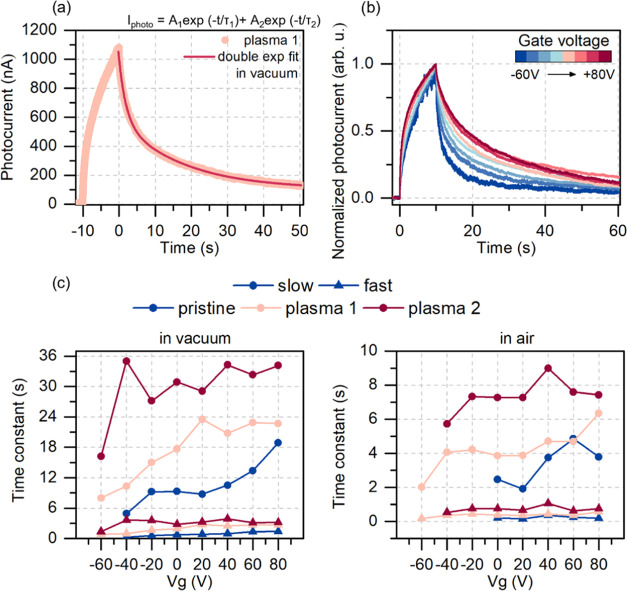
Relaxation time dependence on the gate voltage. (a) The result of fitting a double exponential function to the photocurrent decay signal of the plasma-treated sample. (b) Normalized photocurrent signal of the sample after the first plasma treatment in vacuum showing the effect of the applied gate voltage on the photocurrent relaxation in the sample. The lower the gate voltage applied, the faster the relaxation. This effect is attributed to the occupancy of the recombination centers in the band gap. (c) The slow and fast photoresponse time constants for each sample resulting from the double exponential fit in (a). The slow component shows a stronger dependence on the applied gate voltage. The significant noise for the untreated sample at low voltages in air resulted in the inability to perform the fit and therefore missing points in the plot.

We also found that the plasma treatment influenced the relaxation times of the photocurrent. We fitted the photocurrent decay after the illumination was turned off with a double exponential function,^3,11,21^ which resulted in obtaining two time constants corresponding to fast and slow contribution to the signal (see Figure 4). The fitting formula was , where τ_1_ and τ_2_ are the time constants of the fit and *A*_1_ and *A*_2_ are the amplitudes. After each plasma process at zero gate bias, the photocurrent decay time components almost doubled in the response time in both environments. The considerable photocurrent enhancement comes, therefore, with a cost of a slower photoresponse. Such an exchange would be beneficial for several applications such as UV-enhanced gas sensors, emerging visible light positioning systems not requiring millisecond precision, optoelectronic memories, and synapses to set the information storage time to the desired value. The response time can also be partially controlled by applying a gate voltage, as shown in Figure 4. Low gate voltage applied results in faster relaxation. The decay time increases for high gate voltage applied. Figure 4c shows the time constants obtained at each gate voltage applied. The slow component exhibits a substantial increase in value for each plasma treatment and increases with higher gate voltages. The fast component (usually attributed to the photoconductive effect) roughly doubles in its value after each plasma treatment but does not show such a strong dependence on the gate voltage. The slow component’s gate dependence suggests its relation to trap states in the band gap. Pushing the Fermi level toward the valence band (applying negative gate voltage) results in more unoccupied states within the energy band gap. These states act as additional recombination centers for excited electrons. At high gate voltage with the increasing Fermi level, more and more states are occupied, and therefore, the recombination time is longer, as previously reported.^22^ So, the response time of such a device can be partially controlled by the dielectric gate. The distinction between recombination centers and trap states should be considered because it is the latter that causes the increase of the response time in both components by trapping the photogenerated charge carriers. The reason for the increase of the response time, while simultaneously observing the decrease of the photocurrent after the second plasma treatment, is hypothesized that although the sample with more structural defects can trap the photogenerated electrons for a longer period, the photocurrent generation becomes less effective due to the introduction of too many defects to the crystal structure.

As a result of the plasma treatment, we also noticed an enlargement in the hysteresis of the transfer characteristics of the WS_2_ FET and a shift of the threshold voltage in transfer characteristics under illumination. These changes are shown in Figure S4. The hysteresis results from carrier trapping in TMD layers, either extrinsic (adsorbates) or intrinsic (trap states).^23,24^

The observed outcome of photocurrent enhancement and decay time change could be attributed to several effects and require further discussion. The first expected effect could be an introduction of sulfur vacancies, which will be discussed in more detail in the next paragraph. The second one could be attributed to the creation of TMOs in their stoichiometric and nonstoichiometric forms. Indeed, it was already observed that the oxygen plasma treatment forms a transition metal oxide on the surface of MoS_2_.^25,26^ The mechanism previously attributed to the TMO photocurrent enhancement in MoS_2_ was the formation of MoS_2_–MoO_3–*x*_ junctions, which serve as carrier trapping sites. These randomly formed TMOs in their stoichiometric form have band gaps of ∼3 eV depending on the fabrication method and annealing, for example, MoO_3_ (3.03,^27^ 3.14 eV^28^) and WO_3_ (2.97,^29^ 3.24 eV^30^). Nonstoichiometric WO_3–*x*_ oxides were shown to have lower band gaps (3.1, 2.6 eV) depending on the oxygen pressure in the growth process.^31^ These energies of band gaps are just below the illumination wavelength used in our experiment, which is ∼3.4 eV (365 nm). Both WO_3_ and MoO_3_ in their nonstoichiometric form were shown to generate photocurrent.^31,32^ Thus, the formed oxide in our samples may also be responsible for electron–hole pair generation, contributing to the total photocurrent enhancement. To prove the above hypothesis and compare the versatility of the plasma treatment enhancement on TMDs, we measured the photocurrent on another sample. Here, we repeat the experiment on MoS_2_ instead of the WS_2_-based device under two different wavelengths (365 nm and 533 nm/2.33 eV) to see if the same plasma parameters would also induce such a high photocurrent enhancement as in the previous samples (see Figure S5). Indeed, it was confirmed that the enhancement in UV light was stronger than that in green light, despite the initially almost equal signal values, most likely due to the light energy above the band gap of MoO_3_.

Now, we focus on the environmental impact on the treated samples. The environmental dependence is seen as the difference between the first and second plasma treatments in air and vacuum (Figure 3) and also shows the strong relationship of the photocurrent with environmental adsorbates, suggesting a significant influence on the structural defects with sulfur vacancies being the most common.^33^ Defect sites are the optimal spots for the environmental molecules’ adsorption on the surface of the sample.^14,34,35^ Sulfur vacancies could be introduced in our material by the nonreactive argon plasma, which was half of the gas mixture used in the process. These defects were also shown to cause the occurrence of the additional trap states in the WS_2_ band gap.^36,37^ The adsorbed environmental molecules (O_2_, H_2_O) on the device in the dark limit its electrical performance by trapping the electrons flowing through the channel. Positive gate bias causes oxygen and water adsorption on the sample, whereas these molecules are desorbed at negative gate voltages.^38^ So the performance of the TMD-based FETs in the air in the dark, despite applying high gate voltages and thus increasing the electron density in the channel, is still strongly hindered due to charge trapping in the adsorbed molecules.^38^ Upon illumination, these trapped electrons can recombine with photogenerated holes, resulting in increased current by photogenerated electrons remaining unrecombined in the channel. These photogenerated electrons may be trapped by adsorbate traps and then again recombine with photogenerated holes, which is the reason for a gradual, slow rise of the photocurrent in the time domain until these processes of adsorption and desorption reach equilibrium.^39,40^

The remarkable environment-dependent difference between the first and second plasma treatments confirms the effects of the gate-bias-induced molecules’ adsorption and desorption processes in photogeneration.^38,39^ In the air, under low gate voltage bias, the dominating photocurrent signal was after the second plasma treatment. Low gate voltage applied means that there are still unfilled traps in the band gap and fewer surface adsorbates on the TMD sample. Two plasma processes are likely to result in more sulfur vacancies, leading to the formation of trap states in the gap.^36^ The trap states keep the photogenerated carriers for a longer time, resulting in higher photocurrent and slower time response.^22^ Applying higher gate voltages results in the increase of the Fermi level and filling of trap states, which are attributed to be the main reason for the photocurrent enhancement after the second plasma treatment.^22^ Therefore, the dominating signal becomes the photocurrent after the first plasma treatment.

The lower photocurrent enhancement after the second plasma treatment in vacuum is most likely caused by the effect of too many defects. The sample after the first plasma treatment has the balance of the efficient photocurrent generation of the direct band gap, more stoichiometric TMD (WS_2_) with the small addition of the favorable defect states due to atomic vacancies, heterojunctions with TMO, and TMO itself under UV light. After the second plasma treatment, the sample has even more trap states and even more oxidized areas, which we can observe as the further increase of the relaxation time of the photocurrent. However, the photocurrent generation and current flow of such a sample are reduced because of large numbers of oxide intrusions, which in larger quantities are less effective in terms of photocurrent generation and sample conductivity. The balance between TMD, TMO, and trap states is disturbed, leading to the lowering of the device performance.

**Figure 5:**
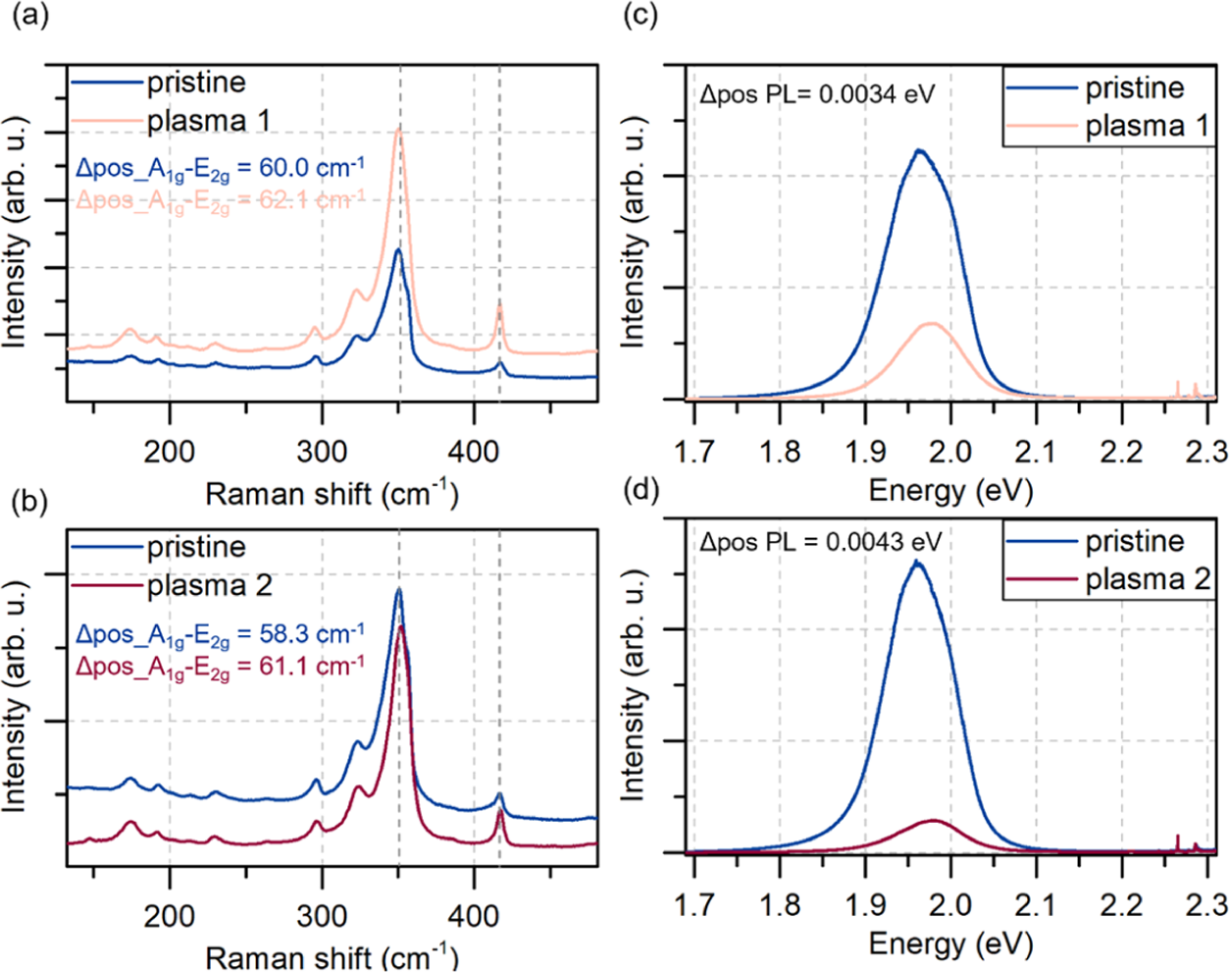
Raman and photoluminescence spectroscopy results. (a, b) Raman spectra of the untreated (pristine) and plasma-treated samples of WS_2_ (one plasma process—a, two plasma processes—b). The treatment of the samples resulted in the asymmetric shift of the two main peaks (upshift of A_1g_, downshift of E^1^_2g_), changing the difference in peaks’ positions (Δpos A_1g_–E_2g_). (c, d) Photoluminescence spectra of WS_2_ untreated and treated samples. The plasma treatment resulted in the signal quenching and the blue shift of the peaks by Δpos PL = 0.0034 eV (c) after the first plasma process and 0.0043 eV (d) after the second plasma process. The position of the peak was calculated as the mean value of the two fitted Lorentzian curves.

The photocurrent enhancement seen in our samples could be attributed to sulfur vacancies (along with the introduced trap states in the band gap), nonstoichiometric TMOs resulting in traps at the formed junction, and stoichiometric TMO formation with the optimal choice of illumination wavelength. To further verify any of the mentioned possibilities, Raman, photoluminescence, and X-ray photoelectron spectroscopy (XPS) spectra were taken on both WS_2_ and MoS_2_ samples before and after the first and second plasma processes. The Raman and photoluminescence average results of the statistical mapping (121 points) for the WS_2_ sample are shown in Figure 5. Similar results for the MoS_2_ sample are shown in Figure S6 in the Supporting Information.

In both TMDs, in Raman spectra, we observed a slight red shift of the E^1^_2g_ peak and a blue shift of A_1g_ peak, increasing the difference in these two peaks’ positions. Such an asymmetric change in the Raman spectrum was previously ascribed to the formation of the TMO on the sample.^12,41,42^ The blue shift of the A_1g_ peak results from p-type doping (by built-in oxygen),^43^ whereas the change of E^1^_2g_ is described as a distortion of the crystal lattice and the change of the out-of-plane vibration of sulfur atoms.^26^ The peaks’ width also changes (their broadening would be expected^26^), but these changes are a bit more challenging to address for WS_2_ due to the occurring resonance at 532 nm (the matter is further described in the Supporting Information, the individual points of the mapping measurement are shown in Figure S7). The plasma treatment was optimized for WS_2_ by repeated measurements at different plasma process settings. For MoS_2_, the treatment was stronger, and the process details are explained in the Supporting Information (see Figures S6 and S7). Still, despite the strong treatment, the enhancement mechanisms occurred in both samples. Both materials undergo structural changes, along with the expected oxidation. The average photoluminescence spectrum of WS_2_ was also quenched, and the peaks blue-shifted, suggesting the random oxide formation.^12,41,42^ Statistical Raman results prove that the observed effect of photocurrent enhancement could not be attributed to removing the surface contaminants only. The observed spectra change significantly with each plasma treatment, and the changes correspond to the statements of our hypothesis.

**Figure 6:**
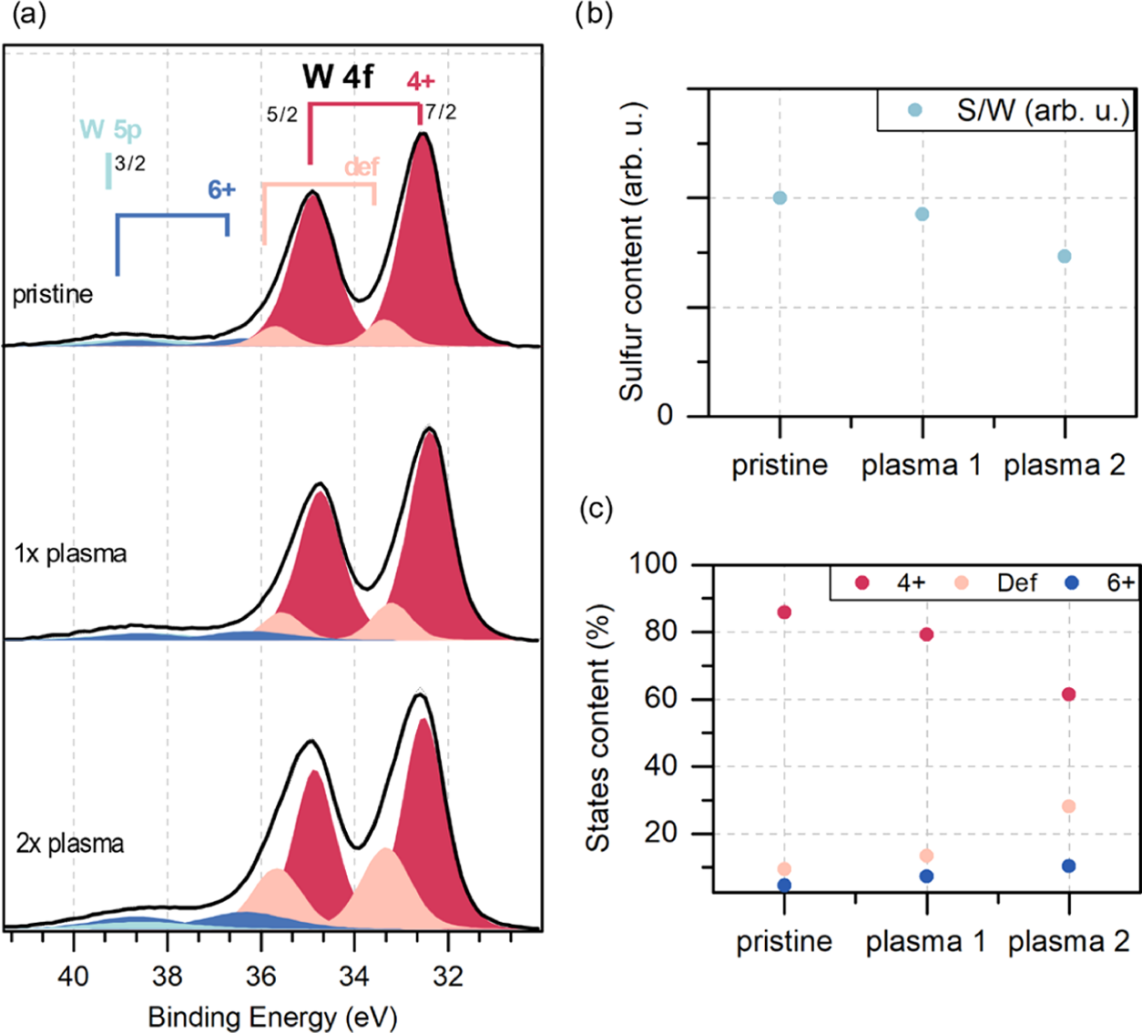
XPS measurements results. (a) XPS spectra with fitted peaks. (b) Relative differences in the S/W ratio of the untreated and plasma-treated samples. (c) Percentages of the individual states in the sample before and after the plasma treatment.

To further support the data from Raman mapping and photoluminescence analysis, XPS spectra were taken on the same samples (WS_2_ is shown in Figure 6 and MoS_2_ is in Figure S8). The XPS measurement of W 4f and S 2p core line spectra allows us to conclude the stoichiometry changes caused by the plasma treatment. The relative differences in the S/W ratio (Figure 6b) indicate that the plasma treatment led to a significant decrease in sulfur content (over 25% after the second plasma treatment). Additionally, the visible changes in the W 4f line shape were analyzed based on the peak fitting procedure. The W 4f region contains a few different species related to different chemical states of W ions, and each state is represented by a spin–orbit doublet line (4f_7/2_ and 4f_5/2_). The main doublet with W 4f_7/2_ maxima near 32.8 eV can be identified as a 4+ oxidation state, indicating the presence of 2H WS_2_.^44,45^ The second doublet shifted to higher binding energy (W 4f_7/2_ line near 36.2 eV) can be identified as coming from a 6+ state present in WO_3._^44,46^ Additionally, the spectrum’s shape requires a third state to be added between the previous two. This state may be related to the presence of nonstoichiometric oxides^47^ or WO_2_;^48^ for the purposes of further discussion, this state will be called the defect state. XPS spectra with fitted peaks are presented in Figure 6a, while the percentages of individual states for the pre- and post-plasma-treated samples are summarized in Figure 6c. The analysis of the WS_2_ sample shows that in each plasma process, there are fewer sulfur S 2p bonds in the material, indicating the occurrence of sulfur vacancies and the possibility of oxide formation. A slight increase of the 6+ peak proves that the traces of WO_3_ can be found on the surface. There is also an intense defect state feature growing with each plasma process that cannot be entirely attributed to any stoichiometric oxides. This indicates that the plasma treatment transformed a part of W–S bonds and led to the formation of nonstoichiometric TMO (WO_3–*x*_) or possibly WO_2_.

The MoS_2_ XPS spectra are shown in Figure S8. The results in MoS_2_ lead to similar conclusions as in WS_2_ samples.

## Conclusion

In summary, we showed an extraordinary on-chip, over 150-fold enhancement of the photocurrent signal and a gradual time response modulation in WS_2_ and MoS_2_ monolayer-based devices by oxygen–argon plasma treatment. The treatment changes the behavior of the samples depending on the environment. The effect can be explained by the co-occurrence of several effects: charge trapping by sulfur vacancies and TMD–TMO heterojunctions, along with effective electron–hole pair generation from the favorable illumination wavelength choice for the excitement of both TMDs and TMO. This method could be used to modulate the photogeneration for the novel applications of the TMDs in optoelectronic applications such as memories, artificial synapses, or others using the effect of persistent photoconductivity or favoring the effects of a strong photocurrent signal over the time of response.

## Methods

### Device Fabrication

The devices were fabricated on the chemical vapor deposition (CVD) samples of WS_2_ and MoS_2_ monolayers on a 300 nm SiO_2_/Si substrate (Sixcarbon Technology, Shenzhen, China). We used the electron-beam lithography technique to fabricate two-terminal FET devices with a bottom gate configuration, a 2 μm long channel, and 5 nm chromium/100 nm thick gold electrodes thermally evaporated.

### Plasma Treatment and Structural Characterization

The plasma process was done using Diener Zepto plasma with an argon–oxygen gas mix in equal proportion. The plasma parameters 4 W, 15 sccm, and 5 s were chosen after a series of optimization measurements. Raman spectroscopy and photoluminescence measurements were done with a 532 nm laser (Renishaw inVia Qontor Raman spectrometer) on the samples after each plasma process. Different samples were used for electrical measurements and spectroscopic measurements.

### Electrical Measurements

Electrical measurements were done using a DL-1211 Current Preamplifier and National Instruments DAQ 6366 with a sampling frequency of 1 kHz. We used Oxford MicrostatHe2 cryostat to provide a vacuum environment for the sample before the measurements for ∼16 h. The pressure was at least 5 × 10^–3^ mbar or lower. For measurements in air, the cryostat was vented, maintaining the cover with a glass window to avoid differences in light scattering. All photocurrent measurements were done applying 5 V source–drain bias. The illumination was provided by a 365 nm LSM diode with an LDC-1 controller (Ocean Insight) for 10 s each. The light power on the sample was 130 μW. For wavelength-dependent measurements, 365 nm and 533 nm LSM diodes were used, operating at an optical power of 30 μW.

### XPS Measurements

The results were supplemented by XPS. The XPS system was equipped with a hemispherical energy analyzer Phoibos 150 (SPECS) with a 2D-CCD detector and a DAR 400 X-ray lamp (Omicron); nonmonochromatic radiation of 1253.64 eV (Mg Kα) was used. The peak fitting procedure was supported by Casa XPS software.

## Supporting Information Available

Photocurrent results obtained for MoS_2_ samples in both environments; showcase of complete results for WS_2_ samples under different gate voltages; transfer characteristics for WS_2_ and MoS_2_ FETs; photocurrent measurements of MoS_2_ under green light illumination; Raman and photoluminescence spectra of the samples before and after plasma treatment; and XPS results of the MoS_2_ sample (PDF)

## Abbreviations

TMDs: transition metal dichalcogenides
WS_2_: tungsten disulfide
MoS_2_: molybdenum disulfide
PPC: persistent photoconductivity
FETs: field-effect transistors
TMO: transition metal oxide
WO_3_: tungsten oxide
MoO_3_: molybdenum oxide
O_2_: oxygen
H_2_O: water
XPS: X-ray photoelectron spectroscopy
CVD: chemical vapor deposition
