# Supplementary material for: Unraveling the Mechanism of the 150-Fold Photocurrent Enhancement in Plasma-Treated 2D TMDs
Source: ACS Appl Mater Interfaces. 2022 Jul 18;14(29):33984–92. doi: 10.1021/acsami.2c06578 (PMC9335404; doi:10.1021/acsami.2c06578)
Supplement: Supplementary file 1 — am2c06578_si_001.pdf [file am2c06578_si_001.pdf]

Supporting Information

Unraveling the Mechanism of the 150-fold Photocurrent Enhancement in Plasma-Treated 2D TMDs

Karolina Czerniak-Łosiewicz[a], Michał Świniarski[a], Arkadiusz P. Gertych[a], Małgorzata Giza[a], Zofia Maj[a], Maciej Rogala[b], Paweł Kowalczyk[b], Mariusz Zdrojek[a]*

[a]Faculty of Physics, Warsaw University of Technology, Koszykowa 75, 00-662, Poland

[b]Faculty of Physics and Applied Informatics, University of Lodz, Pomorska 149/153, 90-236, Poland

*karolina.czerniak@pw.edu.pl

Table of contents

1. MoS_2 results in both environments....2
2. WS_2 sample complete set of results in both environments for all gate voltages applied....3
3. Transfer characteristics of WS_2 and MoS_2-based FETs....4
4. MoS_2 results under different illumination wavelength....6
5. Raman and photoluminescence spectra of MoS_2 and WS_2 before and after plasma treatment....6
6. XPS results for MoS_2....8
7. The comparison between dark and illuminated transfer characteristics....9

1. MoS_2 results in both environments

For comparison with the sample in the main text, the MoS_2 devices were fabricated and studied in the same manner as WS_2. The plasma process was mainly optimized for WS_2 samples, but several attempts have been also made for MoS_2. One such attempt was treating the sample with stronger, and more aggressive 40 W, 5 SCCM, 1 s plasma, which is shown in Figures S1 and S2. For this sample, the environmental dependence of the structural modification was evident. The pristine sample was the one with the lowest photocurrent signal. The sample after the 1st plasma showed the strongest photoresponse in a vacuum, while in the air, it was the 2nd plasma that was the one with the best response in terms of current value. Such parameters were still too strong, resulting in a large signal noise, long photocurrent relaxation, and not as high photocurrent enhancement as in the WS_2 samples described in the main text. Still, they were used for the Raman spectroscopy, photoluminescence, and XPS study to determine what changes occur in the material's structure when the measurement environment influences the photocurrent signal as strongly as in this case.

In a vacuum (Figure S1), we can observe an enhancement of the photocurrent for the sample after the 1st plasma treatment. The 2nd plasma treatment made the signal decrease to a very comparable as in the pristine sample. The plasma treatment also increased the decay time of the photocurrent.

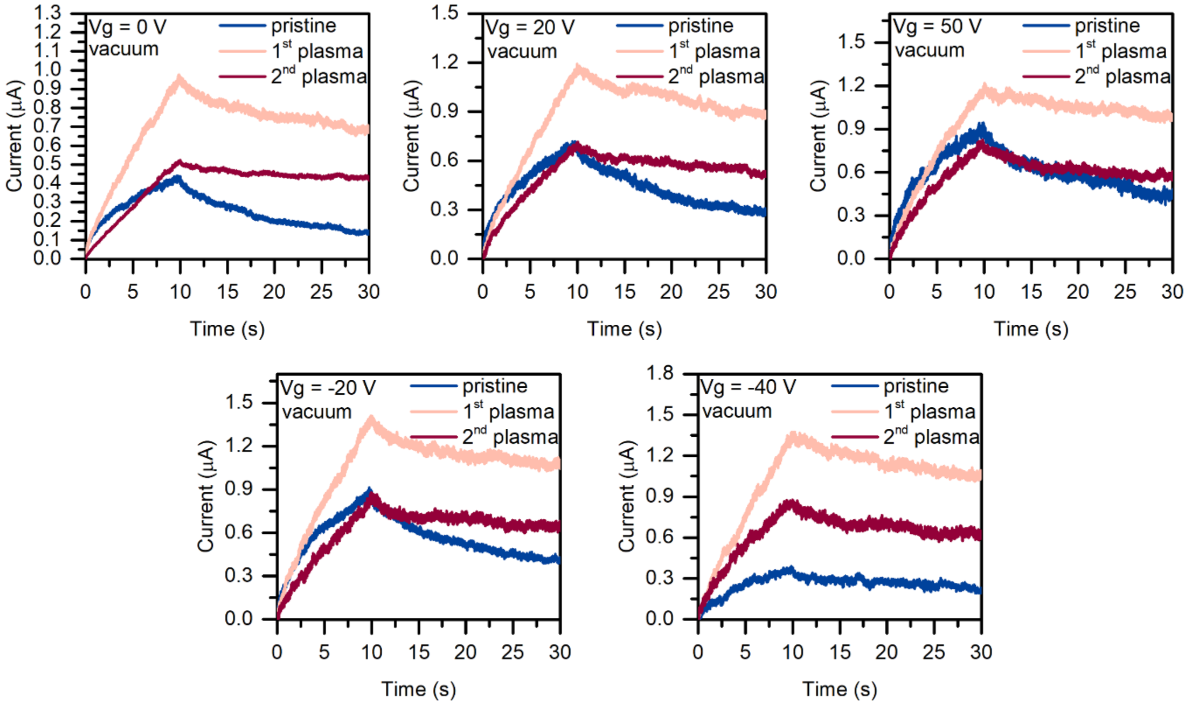

Figure S1 The photocurrent signal of the samples before (pristine – blue lines) and after plasma treatment (1st plasma – beige lines, 2nd plasma – red lines) measured in vacuum. The graphs show the gate voltage dependence of the photocurrent for 0 V, 20 V, 50 V, -20 V, -40 V. Large enhancement of the photocurrent was observed after the 1st plasma treatment. The 2nd plasma treatment also increased the photocurrent signal compared to the pristine sample, but the enhancement effect is not as pronounced as the 1st treatment.

In the air, contrary to the vacuum, the strongest signal comes from the sample after the 2nd plasma treatment, as shown in Figure S2.

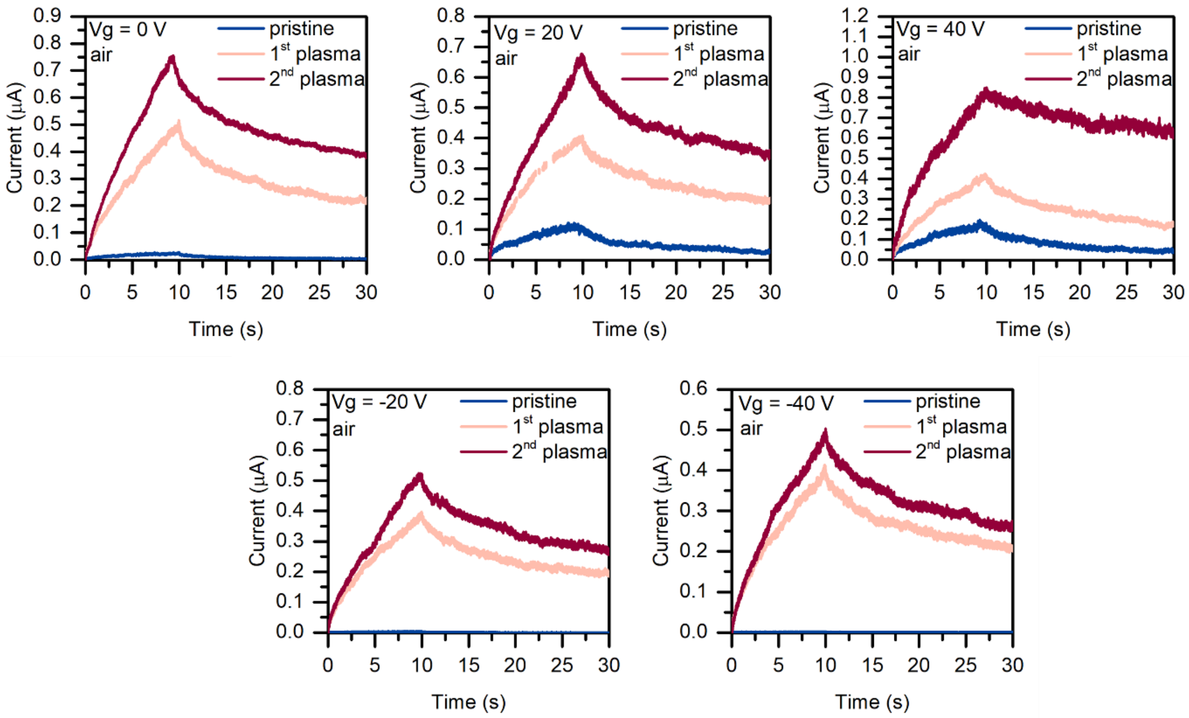

Figure S2 The photocurrent enhancement for the MoS_2 sample in air. The strongest signal is obtained for the sample after two plasma treatments.

The obtained results prove the versatility of the plasma treatment method for photocurrent enhancement which is also environment-dependent.

2. WS_2 sample complete set of results in both environments for all gate voltages applied

To get a full image of the signal changes due to different gate voltages applied and the measurement environment, all time-resolved photocurrent signal plots are shown in Figure S4 below. One of the first things to notice is the gate-dependent behavior between the treated and untreated samples, especially in the air. The pristine sample in the air at high gate voltages does not follow the trend of rising signal in each measurement. Instead, the signal is actually lower for the sample at 80 V than it was for 60 V. This effect is probably related to the adsorption of environmental molecules at high gate voltages[1]. The molecules affect the channel by limiting its conductivity, which can affect the photocurrent generation[2]. After the plasma treatment, the device in air shows a visible distinction between high (40 V – 80 V) gate voltages applied, which is most likely related to the sulfur vacancies adsorbing molecules at defect sites but now promoting the trapping mechanism instead of limiting the conductivity of the channel. Another important observation is the change in the relaxation time of the samples. The photocurrent decay in both environments gets longer with each treatment due to charge trapping at the created traps induced by plasma.

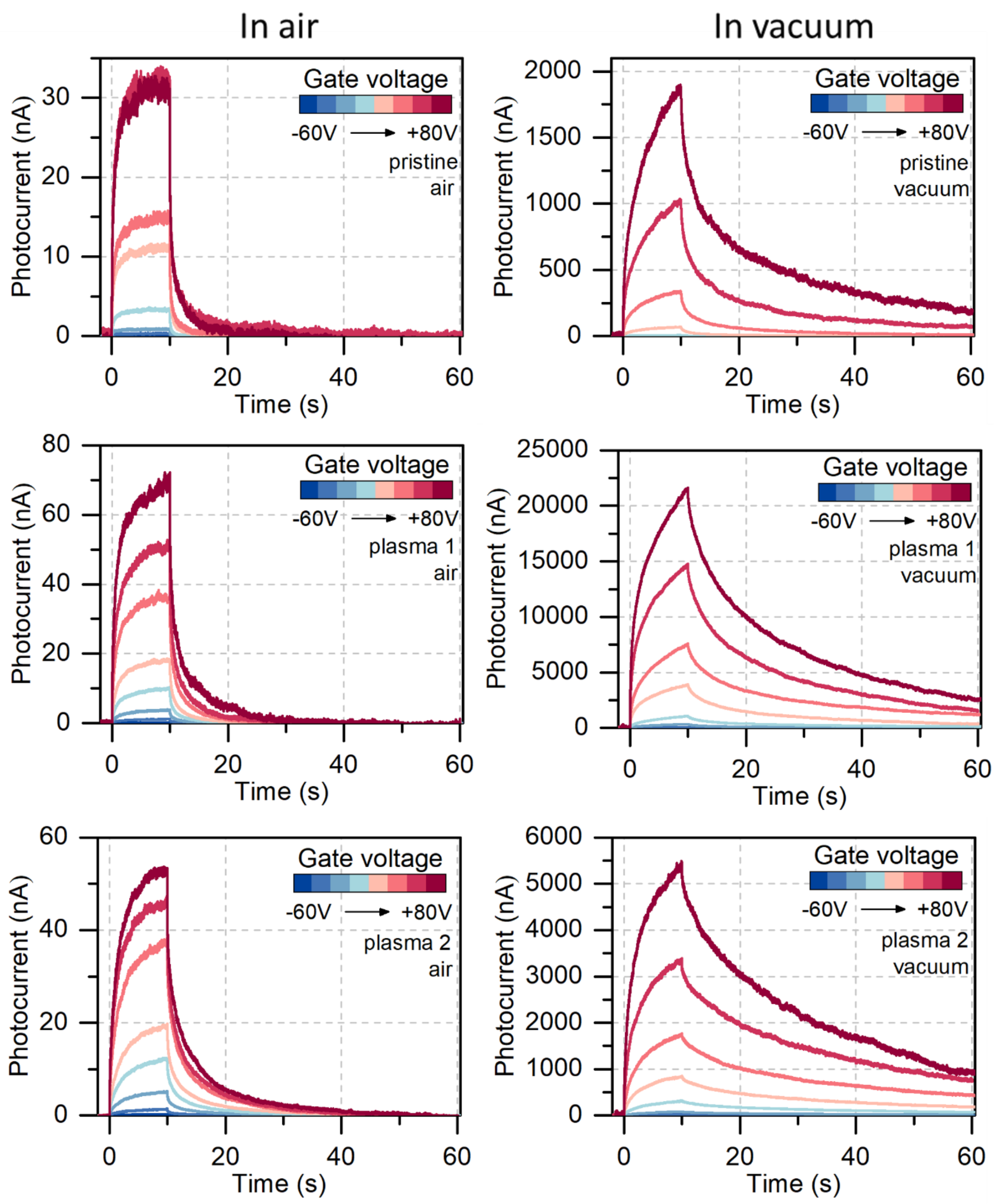

Figure S3 All obtained results for the measured photocurrent in the air (left) and vacuum (right) environment.

3. Transfer characteristics of WS_2 and MoS_2-based FETs

In addition to the photocurrent measurements in this work, the transfer characteristics of the device in the dark and under constant illumination were measured after each step of treatment. The results in Figure S4 show that the hysteresis of the devices, especially in the air, rises as expected from a defected structure[3,4]. In air after the 1st plasma treatment, the current in the dark is lower, whereas it is the highest in either environment under illumination.

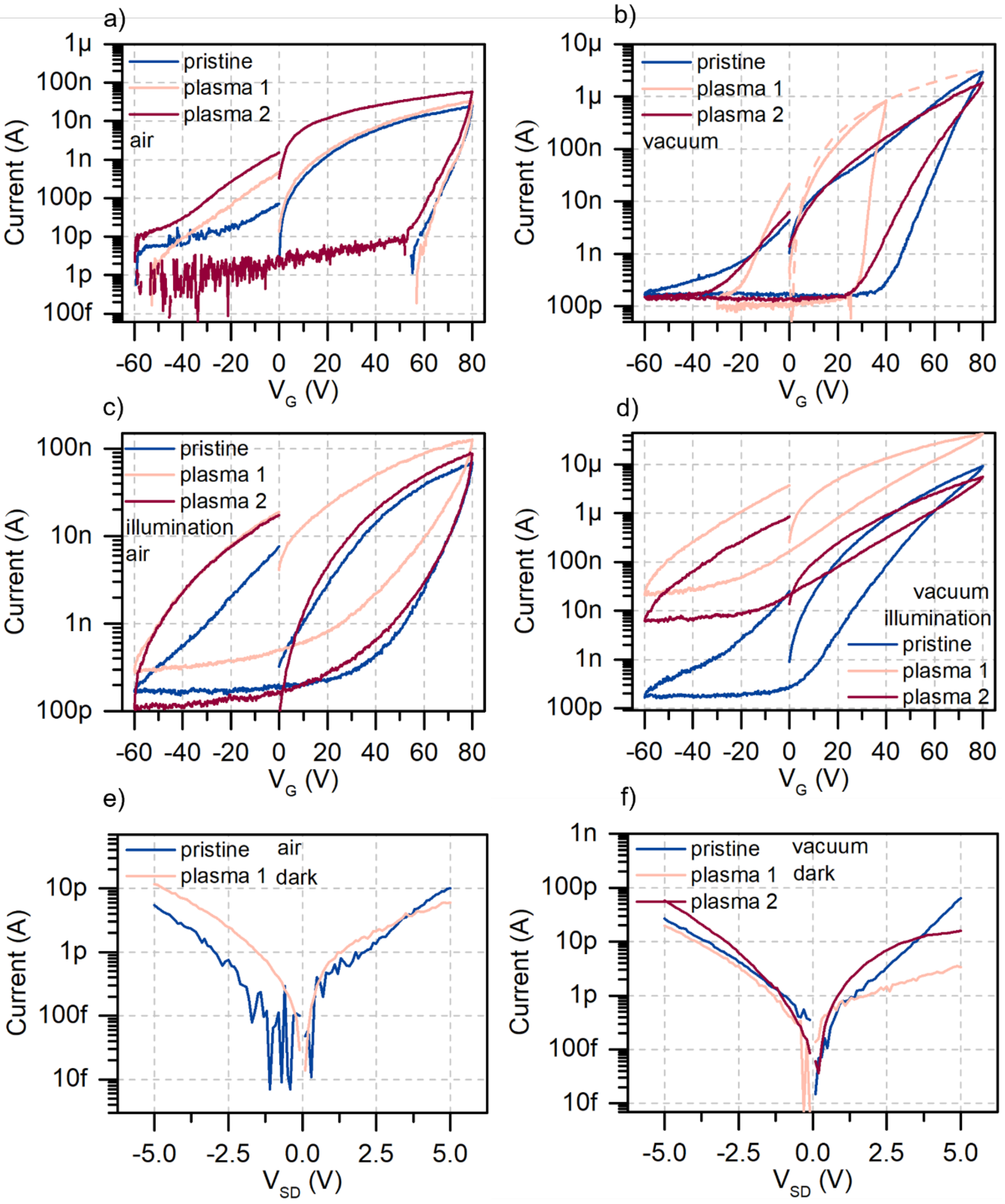

Figure S4 a)-d) Transfer characteristics measured in air and vacuum for the WS_2 sample. e)-f) Output characteristics of the devices measured in air and in vacuum at zero gate bias. The measurement in the air for the 2nd plasma-treated sample was indistinguishable from the measurement noise. The devices show a Schottky barrier, which results in very low dark current – favorable for photocurrent studies.

We note that the Figure S4 b for plasma 1 shows with a dashed line the extrapolation by Schockley's equation $I_D = I_{DSS}\left(1 - \frac{V_G}{V_P}\right)^2$, where I_{DSS} is the drain current at V_G = 0 V, V_P is the gate voltage at drain current I_D = 0 A[5]. The extrapolation was done due to a measurement setting error. The same data were used in Figure S9 to compare dark and illuminated transfer characteristics after each treatment directly.

4. MoS_2 results under different illumination wavelength

To validate the transition metal oxide formation hypothesis after the 1st plasma treatment in TMDs, another sample of MoS_2 was made and treated with the same parameters as the WS_2 sample in the main text. The device's channel was 2 µm long. It was measured at 5 V bias under 365 nm and 533 nm light with the same illumination power. We note that the light power was lower than in the experiments in the main text due to the limitation of the 533 nm LSM diode (~30 µW). Despite the slightly higher absorption coefficient for 365 nm compared to 533 nm[6], the photocurrent signal shows similar values for the untreated pristine sample, as shown in Figure S3. After plasma treatment, we observe a photocurrent enhancement for both green and UV light. The UV enhancement is, however, larger (here, approximately 40 times) than for the green diode (approximately 10 times). Such a difference confirms our attribution of this effect to the TMO formation on the sample. It also shows that both sulfur-based TMDs can be treated to augment the photo-generated signal.

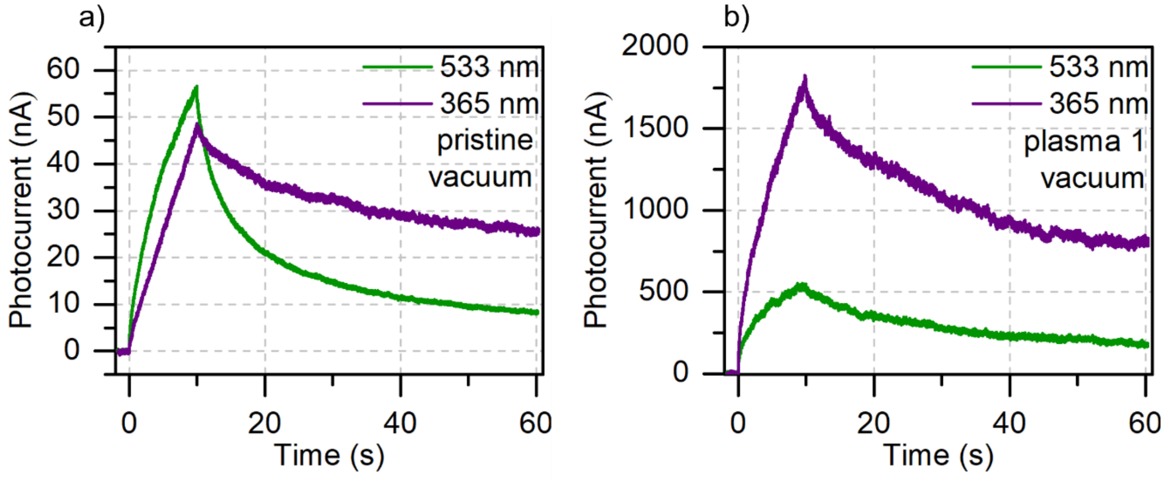

Figure S5 A comparison between the photocurrent obtained from the (a) pristine and plasma-treated sample (b) under different illumination wavelengths. Despite the initially comparable signal for the two wavelengths, the sample shows a larger enhancement under UV light (365 nm), which we attribute to the favorable choice of wavelength for photocurrent generation in both MoS_2 and MoO_3/MoO_{3-x}.

5. Raman and photoluminescence spectra of MoS_2 and WS_2 before and after plasma treatment

The MoS_2 sample treated with stronger, more aggressive plasma than the WS_2 sample from the main text was also subjected to the structural characterization. It was necessary to see what structural changes occurred in the sample after such strong plasma treatment. The two samples were measured spectroscopically as pristine ones, and then the first one of them was treated once and the other twice with strong plasma parameters. The Raman and photoluminescence spectra of the sample (Figure S6) show the same, but more intense results as the WS_2 lightly treated sample. We observed the frequently reported broadening of the Raman peaks due to the impact of the plasma on the crystal lattice[7]. The asymmetric Raman peak shift was also visible in these results. The photoluminescence peak quenched and blueshifted. All these observations suggest that the sample underwent partially an oxide formation on the surface due to oxygen-containing gas mixture in plasma and structural degradation (e. g. sulfur vacancies) due to argon plasma, which is observed as the drop in intensity.

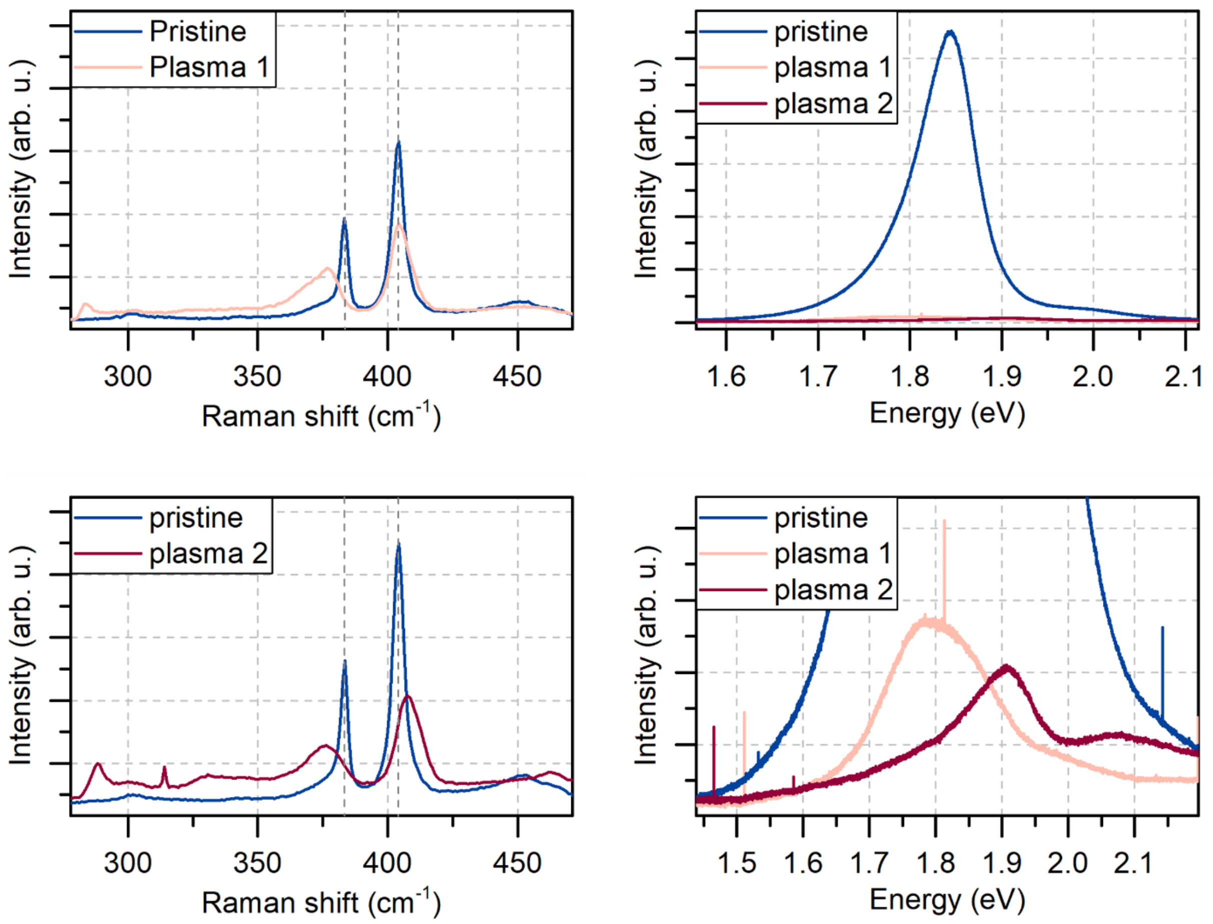

Figure S6 Raman (left) and photoluminescence (right) spectra of the MoS_2 before and after plasma treatment. We observe a significant blueshift of the A_{1g} peak in Raman and photoluminescence peak along with significant quenching.

It is worth noting that the spectra shown in Figure S6 as well as Figure 5 in the main text are the averaged spectra of 121 mapping measurements on the sample's surface (50 μm x 50 μm area). To visualize well the structural changes occurring in the samples, the individual points corresponding to peak positions and width in correlation are shown in Figure S7 below. The images show that the changes are more pronounced for the MoS_2 sample, treated with stronger plasma parameters. The well-optimized parameters of WS_2 plasma resulted in less intense, however, still evident changes in the spectrum. An essential factor in this analysis is also the laser wavelength 532 nm. For MoS_2, the only peaks fitted with Lorentzian functions were E_{2g} and A_{1g}, which were well pronounced. WS_2 illumination with 532 nm results in a resonance spectrum, in which multiple peaks become visible. The E_{2g} peak is a part of a combination of peaks, including highly intensive 2LA, which may result in worse fit quality. A_{1g} at the same time is much less intense than the prominent, combined feature due to the resonance. It also can influence the observed variation of the fitted peaks and result in for example, no evident broadening of the peaks. Nevertheless, the average spectrum and the presented correlations show that the samples undergo structural damages and partially transition metal oxide formation, which is also supplemented by XPS results.

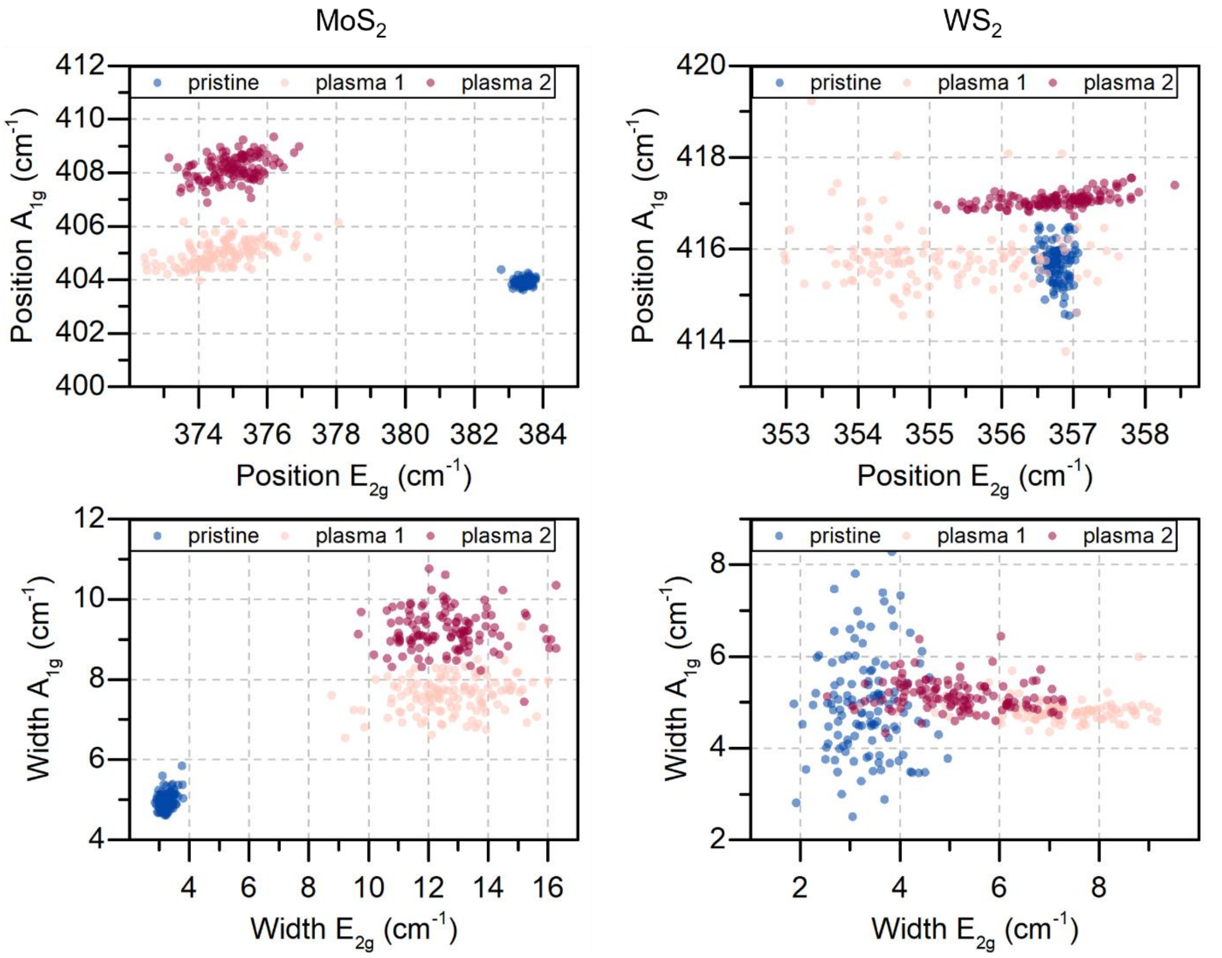

Figure S7 Raman peak positions and widths correlation from 121 points WS_2 (right) and MoS_2 (left) mapping.

6. XPS results for MoS_2

The spectra show similar behavior to the ones observed for the WS_2 sample. The pristine flakes, which are initially slightly sulfur-deficient, probably due to the growth process[8], are further modified by plasma treatment. After 1st and 2nd etching, the sulfur concentration decrease. This is accompanied by the changes in the shape of Mo 3d line caused by the increasing concentration of 5+ and 6+ Mo oxidation states[9,10]. However, in MoS_2, the amount of stochiometric oxide decreases after the 2nd plasma process, resulting from the argon bombardment. Argon plasma acting randomly just as oxygen plasma in equal proportion on the sample could destroy some of the formed MoO_3 structures. The obtained results suggest that the plasma treatment led to the disturbance of the initial stoichiometry of the flakes and the substitution of part of Mo-S bonds, however it is far from a complete transformation to MoO_3[11].

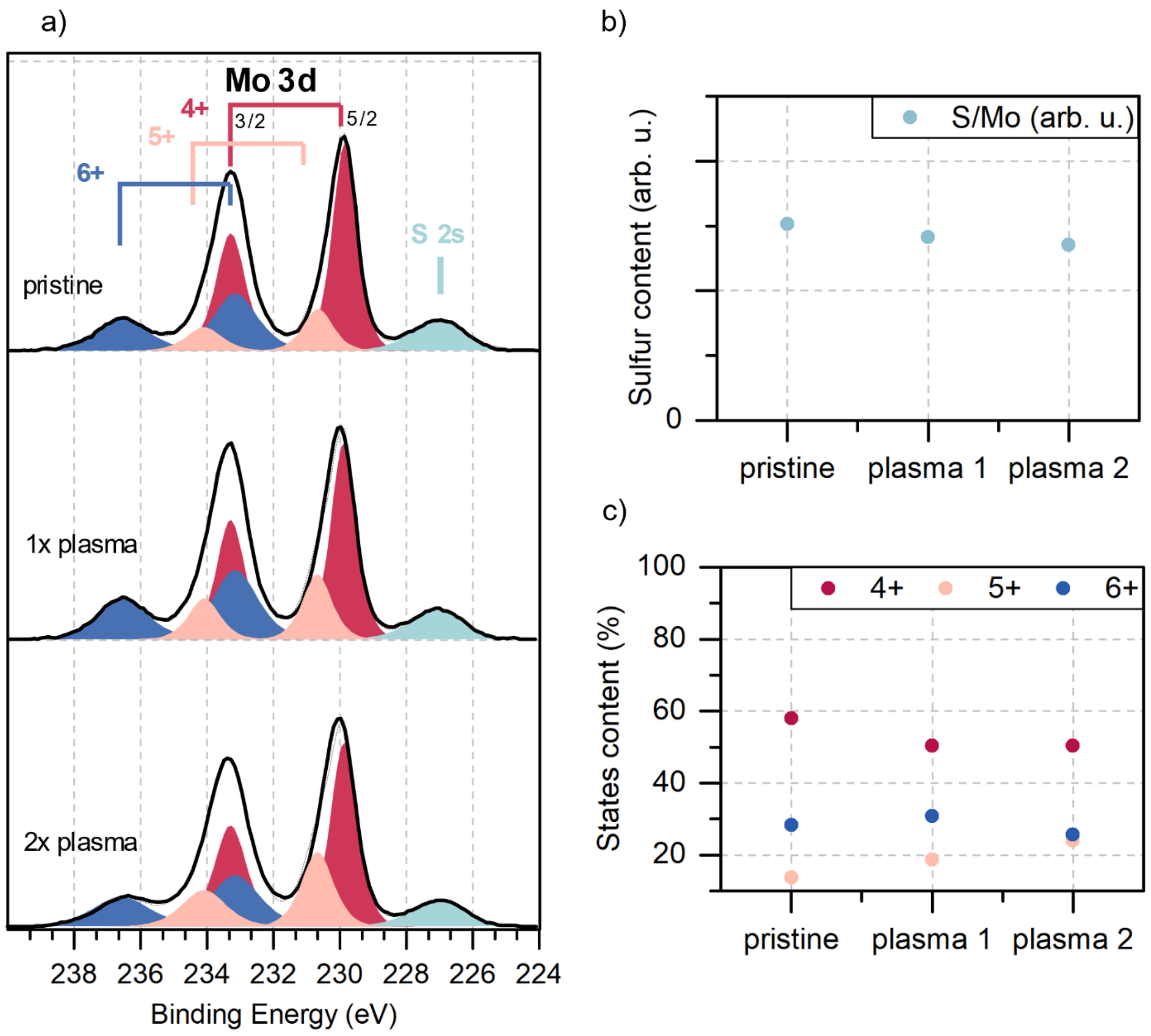

Figure S8 a) XPS spectra of MoS_2 sample before and after 1st and 2nd plasma treatment. b) Sulfur content calculated from the XPS. c) States content in the MoS_2 before and after plasma treatment.

7. The comparison between dark and illuminated transfer characteristics

For a clear comparison, we presented the dark transfer characteristics shown in Figure S4 separately for each plasma process and paired them with the corresponding transfer characteristics under illumination. Such a presentation directly shows the effect of photogating on the treated and untreated WS_2 samples.

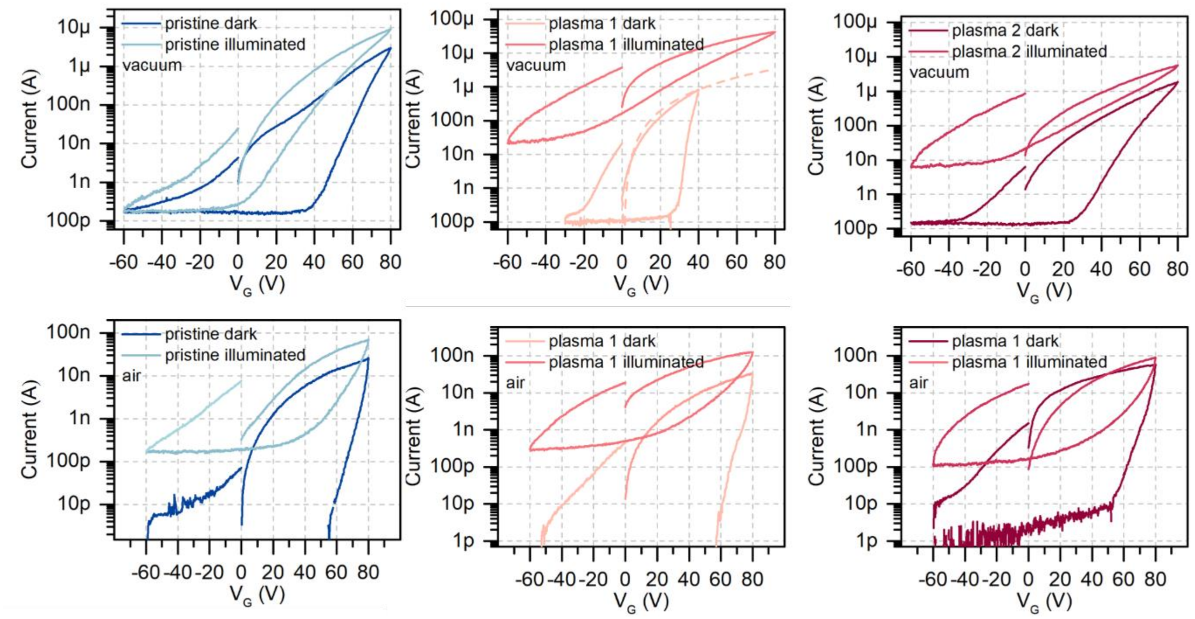

Figure S9 A comparison between dark and illuminated transfer characteristics for pristine and plasma-treated samples in air and vacuum. The graphs correspond to transfer characteristics shown in Figure S4.

8. References

(1) Cho, K.; Park, W.; Park, J.; Jeong, H.; Jang, J.; Kim, T. Electric Stress-Induced Threshold Voltage Instability of Multilayer MoS 2 Field E Ff Ect Transistors. **2013**, No. 9, 7751–7758.

(2) Buscema, M.; Island, J. O.; Groenendijk, D. J.; Blanter, S. I.; Steele, G. A.; Van Der Zant, H. S. J.; Castellanos-Gomez, A. Photocurrent Generation with Two-Dimensional van Der Waals Semiconductors. *Chem. Soc. Rev.* **2015**, *44* (11), 3691–3718. https://doi.org/10.1039/c5cs00106d.

(3) Late, D. J.; Liu, B.; Matte, H. S. S. R.; Dravid, V. P.; Rao, C. N. R. Hysteresis in Single-Layer MoS 2 Field Effect Transistors. *ACS Nano* **2012**, *6* (6), 5635–5641. https://doi.org/10.1021/nn301572c.

(4) Ahn, J. H.; Parkin, W. M.; Naylor, C. H.; Johnson, A. T. C.; Drndić, M. Ambient Effects on Electrical Characteristics of CVD-Grown Monolayer MoS2 Field-Effect Transistors. *Sci. Rep.* **2017**, *7* (1), 1–9. https://doi.org/10.1038/s41598-017-04350-z.

(5) Sze, S. M.; Ng, K. K. Physics of Semiconductor Devices. In *Physics of Semiconductor Devices*; John Wiley & Sons, Inc.: New Jersey, 2007.

(6) Wang, L.; Wang, Z.; Wang, H. Y.; Grinblat, G.; Huang, Y. L.; Wang, D.; Ye, X. H.; Li, X. Bin; Bao, Q.; Wee, A. S.; Maier, S. A.; Chen, Q. D.; Zhong, M. L.; Qiu, C. W.; Sun, H. B. Slow Cooling and Efficient Extraction of C-Exciton Hot Carriers in MoS2 Monolayer. *Nat. Commun.* **2017**, *8*. https://doi.org/10.1038/ncomms13906.

(7) Choudhary, N.; Islam, M. R.; Kang, N.; Tetard, L.; Jung, Y.; Khondaker, S. I. Two-Dimensional Lateral Heterojunction through Bandgap Engineering of MoS2 via Oxygen Plasma. *J. Phys. Condens. Matter* **2016**, *28* (36). https://doi.org/10.1088/0953-8984/28/36/364002.

(8) Ho, Y. T.; Ma, C. H.; Luong, T. T.; Wei, L. L.; Yen, T. C.; Hsu, W. T.; Chang, W. H.; Chu, Y. C.; Tu, Y. Y.; Pande, K. P.; Chang, E. Y. Layered MoS2 Grown on c - Sapphire by Pulsed Laser Deposition. *Phys. Status Solidi - Rapid Res. Lett.* **2015**, *9* (3), 187–191. https://doi.org/10.1002/pssr.201409561.

(9) Chen, Y.; Zhang, G.; Ji, Q.; Liu, H.; Qu, J. Triggering of Low-Valence Molybdenum in Multiphasic MoS2 for Effective Reactive Oxygen Species Output in Catalytic Fenton-like Reactions. *ACS Appl. Mater. Interfaces* **2019**, *11* (30), 26781–26788. https://doi.org/10.1021/acsami.9b05978.

(10) Kibsgaard, J.; Chen, Z.; Reinecke, B. N.; Jaramillo, T. F. Engineering the Surface Structure of MoS 2 ToÂ Preferentially Expose Active Edge Sites ForÂ Electrocatalysis. *Nat. Mater.* **2012**, *11* (11), 963–969. https://doi.org/10.1038/nmat3439.

(11) Szoszkiewicz, R.; Rogala, M.; Dąbrowski, P. Surface-Bound and Volatile Mo Oxides Produced During Oxidation of Single MoS2 Crystals in Air and High Relative Humidity. *Materials (Basel).* **2020**, *13* (14), 3067. https://doi.org/10.3390/ma13143067.
